# Partial sensory rhizotomy in therapy-refractory and recurrent trigeminal neuralgia – a single center experience

**DOI:** 10.1007/s00701-026-06800-y

**Published:** 2026-02-18

**Authors:** Ina Lange, Ehab El Refaee, Marc Matthes, Henry W. S. Schroeder, Jörg Baldauf

**Affiliations:** 1 Department of Neurosurgery, University Medicine Greifswald, Greifswald, Germany; 2 Department of Neurosurgery, Cairo University, Cairo, Egypt

**Keywords:** Trigeminal neuralgia, Therapy-refractory trigeminal neuralgia, Partial sensory rhizotomy, Multiple sclerosis, Trigeminal nerve, Microvascular decompression

## Abstract

**Background:**

Partial sensory rhizotomy (PSR) is an “ultima ratio” procedure for patients with therapy-refractory trigeminal neuralgia (TN). The treatment can be offered to patients without a neurovascular conflict or to patients who did not benefit either from previous microvascular decompression (MVD) or from other interventional procedures. This study presents our experience with PSR.

**Methods:**

Our prospectively maintained database was searched for patients who underwent PSR. We conducted a retrospective analysis of all patients with PSR. Clinical data, MR imaging, surgical videos, and OR notes were evaluated and a telephone interview for the last follow-up was done.

**Results:**

Our search revealed 48 patients treated with PSR between 2004 and 2023. The average age was 59.4 years. Mean history of symptoms was 7.81 years (1–30 years). All types of previous treatments were included. Fifteen patients suffered from multiple sclerosis. A total pain relief was observed in 42 patients immediately after PSR, two patients had a partial pain improvement, and four patients observed no difference. An expected, variable hypesthesia occurred in 37 patients. The most common procedure was a PSR of the lower third. The mean follow-up was 38 months (3–183 months), five patients were lost to follow-up. 28 patients still had complete, 13 partial pain relief. Ten patients still needed medications but were satisfied with a lower dose and generally improved or were even pain-free. Thirteen patients who had benefited from the PSR initially, reported recurrent TN. Five of them received a second PSR after 5–55 months with complete pain relief.

**Conclusion:**

Early after surgery patient satisfaction regarding pain relief/improvement was 92.7% (44 of 48 patients). During follow-up we observed complete or partial pain relief in 31 of 43 patients (72.1%) without pain medication, another 23.3% were satisfied with on-going medication. However, the degree of sensitive deficits is not predictable. Because of our convincing results, patients should be informed about PSR as a therapeutic option for therapy-refractory or recurrent TN. It may be considered either instead of, or as an alternative to, percutaneous procedures or radiosurgery.

## Introduction

Trigeminal neuralgia (TN) is known to be one of the most frequent chronic neuralgic pain syndromes which can significantly reduce the quality of life [21]. The general incidence is about 4–5/100.000 with higher incidence in older people [23, 33]. It is characterized by short, shock-like, lancinating, and usually one-sided facial pain attacks of most severe intensity. Triggers are usually meaningless events such as chewing, talking, touching, teeth brushing, or cold temperatures [43].

The most likely cause is focal demyelination of the trigeminal nerve at the root entry zone (REZ) [24]. Vascular compression by an artery, or less frequently by a vein, is generally accepted as the main cause for TN, at least since Peter Jannetta's report in 1993. But even Jannetta noted one exception, a TN caused by multiple sclerosis (MS) [22]. In contrast to classical TN, a symptomatic or atypical form is thought to be caused by multiple sclerosis-related demyelinating plaques at the REZ [14, 36]. These patients are usually younger, and the proportion of bilateral TN is significantly higher [30]. If no cause is specifically identified, it is referred to as idiopathic TN.

Although medical treatment remains the preferred therapeutical standard in TN, there are various percutaneous, radiosurgical, and surgical therapy options [5, 6, 31]. The standard surgical procedure if a neurovascular conflict exists is microvascular decompression (MVD). However, if there is no vascular compression, MVD is not indicated. Beside radiosurgery, percutaneous treatment includes glycerol injections, thermocoagulation, and balloon compression [19, 34].

A challenge is the recurrence after these procedures, even after the apparently causal therapy by using MVD or in patients without a neurovascular conflict [27]. Bederson and Wilson described a recurrence rate of 2% per year after initially successful MVD [4]. In the literature, a recurrence rate after percutaneous procedures of up to 80% has been reported [42]. A prolonged duration of symptoms appears to adversely affect the success rate of MVD [3, 9].

If MVD or percutaneous procedures fail, partial sensory rhizotomy (PSR) is an option for therapy-refractory TN. In 1929, Dandy published his first case series of treating TN with partial transection of 50–60% of the trigeminal nerve for the first time through a suboccipital approach [11, 12]. However, the literature describes PSR as having a higher complication rate and less favorable outcome. The postoperative loss of sensitivity may further reduce satisfaction despite pain reduction. Zakrzewska et al. mentioned that patients with PSR are less satisfied than patients with MVD [44]. Notably, Bederson and Wilson reported a 10–20% treatment failure rate after PSR, with an average follow-up of 5.1 years [4].

The aim of this study was to report and review our experience with PSR in patients with therapy-refractory TN.

## Material and methods

Our trigeminal neuralgia database has been prospectively maintained since 2004. We retrospectively reviewed this database for patients with therapy-refractory TN who were treated with PSR at our institution between 2004 and 2023. Clinical data, MR imaging, surgical videos, and OR notes were evaluated and a telephone interview for the last follow-up was done.

All patients had received other treatment options before with poor or no improvement of the symptoms. We have included all previous treatment options. Patients were informed about radiosurgery and percutaneous procedures but preferred PSR after detailed informing about pros and cons of the treatment options. During the consultation, it was explicitly pointed out that PSR would not be performed in the event of a clear microvascular conflict.

**Fig. 1:**
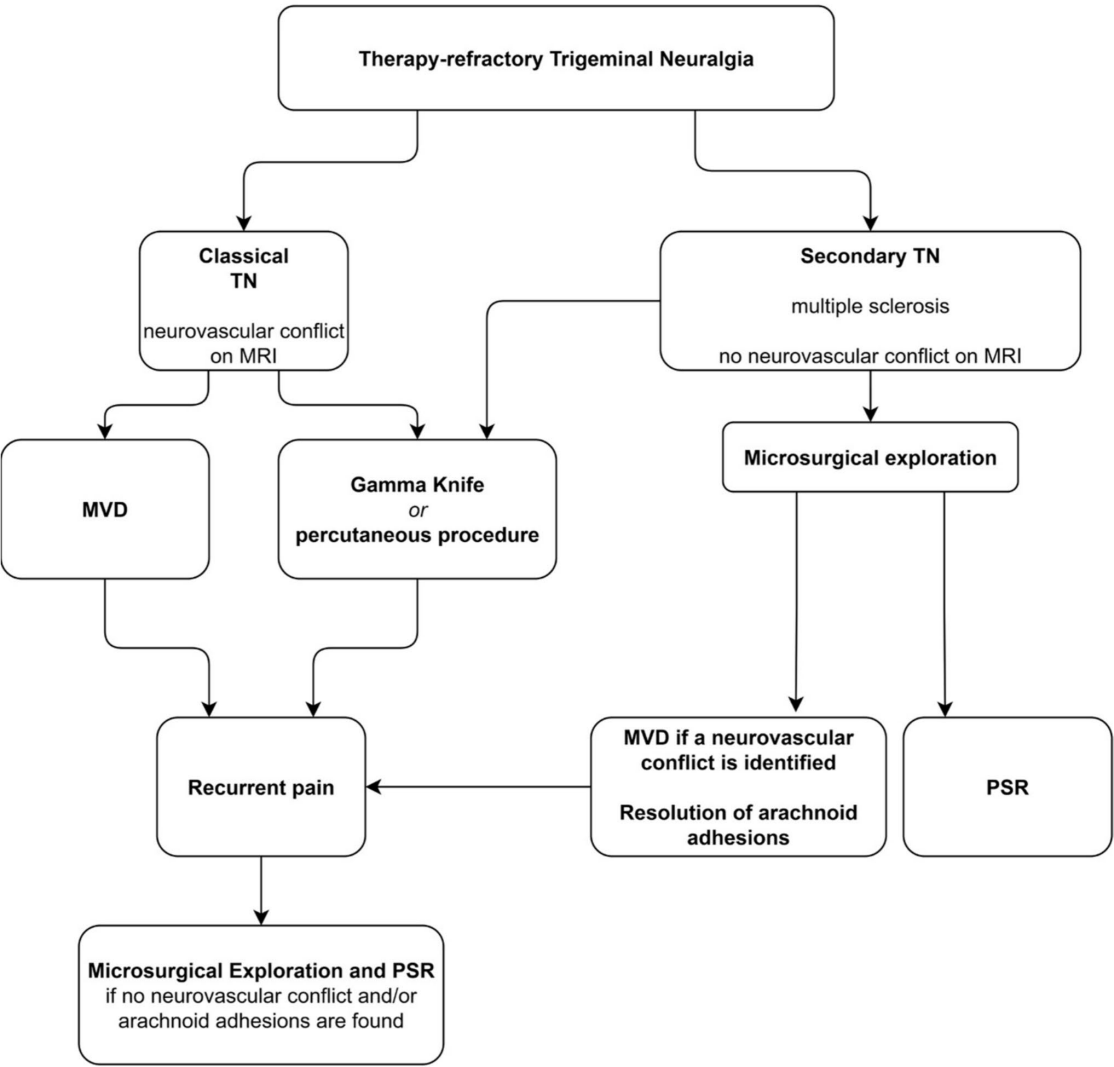
Flow chart for the management of recurrent or therapy-refractory TN leading to PSR

If no compression had been detected, a PSR was done. In a similar management approach, microsurgical exploration and PSR were offered as the initial surgical option in MS patients with no evidence of neurovascular compression on MR imaging. A classical microvascular decompression was not feasible. Because the patients were not satisfied with conservative treatment, microsurgical exploration and PSR were offered. Alternative (percutaneous) treatment options were discussed. However, in the absence of neurovascular compression on intraoperative inspection, patients explicitly opted for PSR rather than any other intervention. If a microvascular conflict or, for example, arachnoid adhesions were identified intraoperatively, PSR was not performed, and these patients were excluded from the present analysis. All patients gave their informed consent. Figure 1 illustrates the decision-making process that ultimately resulted in PSR.

### Surgical technique

**Fig. 2:**
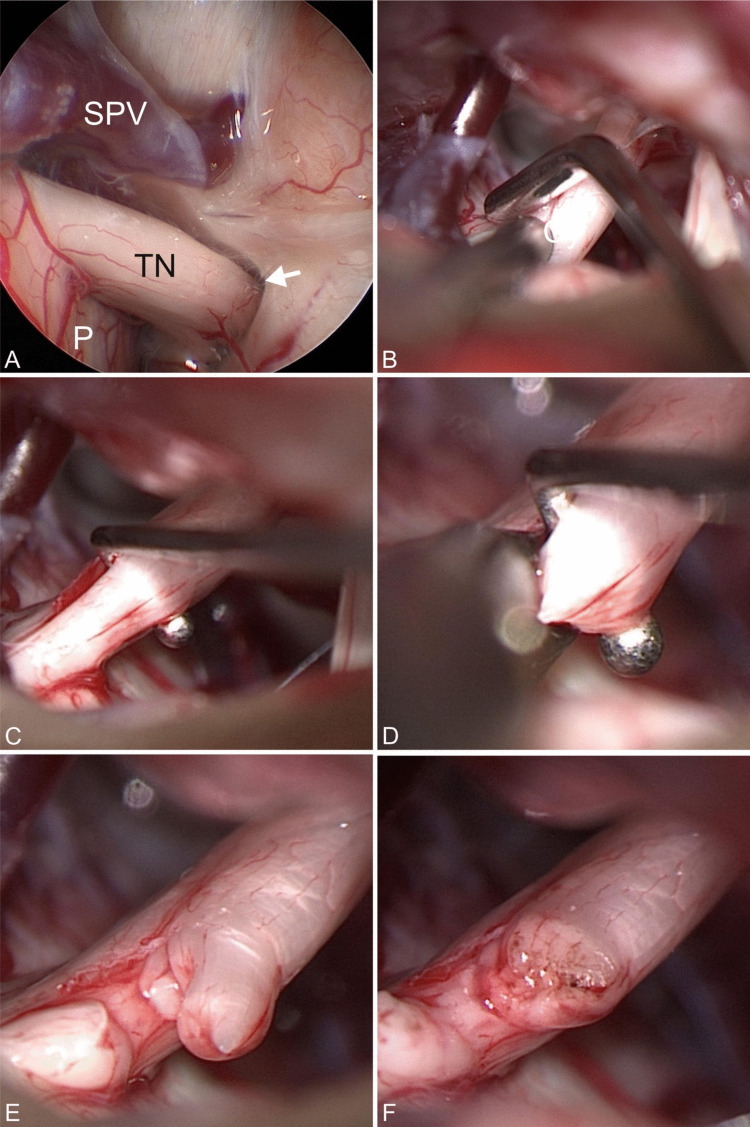
Surgical steps. (**A**) Inspection of the cisternal course of the trigeminal nerve (TN) from pons (P) to Meckel ‘s cave (arrow) with the aid of a 30° endoscope. A contact to the superior petrosal vein (SPV) could be definitively ruled out. (**B**) A nerve stimulator is used to identify the motoric fibers. (**C**) A hook is used to separate the lower one third of the sensory root that is to be severed. (**D**) Cutting of the nerve with scissors. (**E**) View after rhizotomy of the lower third. (**F**) View after coagulation of the nerve endings

The patient was placed in supine position with the head in sharp fixation turned 45° to the opposite side. Monitoring with facial and trigeminal nerve electromyography as well as acoustic evoked potentials was routinely applied. A straight skin incision and dissection of muscles was performed, followed by a superior retrosigmoid (re-) craniotomy of approx. 2 × 2 cm. The dura was then opened in an arc parallel to the sinus knee. In the angle between petrous bone and tentorium, the trigeminal cistern was approached. Previous surgery/MVD required careful dissection of newly developed arachnoid adhesions and inspection of the former MVD complex. An endoscopic inspection was routinely performed before any further surgical step to get a more detailed information of the surrounding anatomical conditions (Fig. 2A). Most of the procedure was performed under the view of the operating microscope. In all patients with a history of MVD, the implanted Teflon was removed.

After confirming the absence of any other offending vessel suitable for MVD, the motor and sensory fibers of the trigeminal nerve were identified using endoscopic inspection and intraoperative neuromonitoring (IONM). Electric trigeminal nerve stimulation was done to clearly identify the motor fibers (Fig. 2B.). After identification, the sensory root had to be visually divided into three parts according to the branches. With respect to the individual pain distribution the amount of fibers was estimated, separated (Fig. 2C) and cut using microscissors (Fig. 2D and E). The separated ends of the nerve were shrinked by gentle coagulation (Fig. 2F). The first branch of the sensory trigeminal nerve must always be preserved to avoid neurotrophic keratitis.

### Outcome measures

The pain was assessed according to the Barrow Neurological Institute (BNI) Score for facial pain preoperatively and during follow-up [39]. The BNI is a 5-level score to classify the intensity of facial pain. Additionally, the BNI score for facial numbness, a 4-level score, was used to assess postoperative hypesthesia [39].

Any deviation from a regular postoperative status was recorded as a complication.

### Statistical analysis

All statistical analyses were performed using SAS version 9.3 (SAS Institute, USA). Group comparisons for categorical variables were carried out using Fisher’s exact test. A p-value of < 0.05 was considered statistically significant. Patients lost to follow up were excluded from outcome-related analyses.

## Results

### Patient demographics and baseline characteristics

48 patients with unilateral TN were included. The average age was 59.4 years (range: 33–82 years). The mean duration of symptoms was 7.81 years (range: 1–30 years). 26 patients were female and 22 were male. The demographic data are shown in Table 1.

**Table 1:** Demographic data

	Total	Male	female
Number of patients	48	22	26
Age (years)	59.4	59.4	59.2
Symptoms			
Typical	36	18	18
Atypical	3	0	3
Combination	9	4	5
Side			
left	28	13	15
right	20	9	11
Affected branch			
V3	24	11	13
V2 + V3	16	9	7
V2	4	1	3
V2 + V1	4	1	3
Pre-PSR treatment			
MVD	22	11	11
Radiosurgery	5	3	2
Thermocoagulation	6	3	3
Glycerol injection	6	2	4
Comorbidities			
Multiple sclerosis	15	9	6
Previous tumor surgery	2	0	2
Duration of symptoms in years	7.8	8.1	7.6

The symptoms were mostly described as typical short but frequent pain attacks which lasted from a few seconds up to a few minutes (36 patients, typical TN or type 1). Twelve patients described a subliminal persistent pain in combination with short typical attacks (atypical TN or type 2).

All patients complained of therapy-refractory TN despite conservative and/or previous invasive treatment respectively. 35 patients did receive either percutaneous, radiosurgical, or MVD treatment besides treatment with medications. Of these, 22 had previous MVD, five patients received radiosurgery and thermocoagulation and glycerol injections were done in six patients each. Some patients received several therapies. All pre-treatments are listed in Table 1.

There were 15 patients with an unknown cause for TN, as there was neither an intraoperative microvascular conflict allowing MVD nor confirmed multiple sclerosis. PSR was then the first surgical approach. Only four of them were pretreated with glycerol injection, but reported insufficient pain relief or recurrent pain.

The mostly affected branch was V3 followed by a combination of the second and third branch (ratio 24:16). In four patients, the second branch was affected. Another four patients had symptoms in V1 and V2.

Before surgery, three patients reported controlled pain with medication (BNI score 3). However, these patients suffered from side effects because of a high dose of medical therapy and requested surgical treatment. 30 patients described not adequately controlled pain despite medication (BNI score 4) and 15 had a severe pain or no pain relief at all under medication (BNI score 5) (Table 3).

Most common was a PSR of the lower third (29), corresponding to the most affected branch. Approximately 50% of the sensory root was cut five times and 2/3 in 11 patients. Three patients received PSR of the middle part of the sensory fibers.

A total of 53 PSRs were performed in 48 patients, with five patients receiving a second PSR during follow-up for recurrent trigeminal neuralgia.

## Outcome

### Short-term postoperative outcome

All patients undergoing PSR were asked daily about their symptoms during their hospital stay of four to five days after surgery. 42 patients described immediate pain relief after surgery and two reported at least 50% or more improvement. Ten patients with immediate postoperative complete pain relief were still on medication at discharge. No significant improvement was reported by four patients immediately after surgery (8.33%).

37 patients described immediate postoperative hypesthesia (77.1%). Two were bothered by this deficit. Extent of sensory loss was very variable as expected. Once we found a slightly weak corneal reflex with complete eye closure and good humidity of the eye. Surprisingly, eleven patients negated any hypesthesia directly after surgery. This applied to eight patients who had a PSR of the lower third, but also two patients after PSR of the middle third and even one patient with a rhizotomy of the lower 2/3.

### Complications

No major complications were observed. Four patients needed further treatment because of cerebrospinal fluid (CSF) fistula. Temporary lumbar CSF drainage was necessary in three patients. Surgical revision was performed in one patient. Two patients reported transient hearing impairment. After inspection by an ENT physician the patients were diagnosed with tympanic effusion. Both recovered within four weeks with full rehabilitation of hearing capacity. Two patients with MS had complications because of their primary disease. One patient with known dysphagia suffered from postoperative pneumonia because of aspiration, another had an MS-related episode. Both recovered fully after specific treatment. The gross complication rate for 53 procedures was 15.1%. Besides expected hypesthesia no permanent deficits were found in any patient. The complications are listed in Table 2.

**Table 2:** Immediate postoperative complications

Complication	Number of patients
CSF fistula	4
Transient hearing impairment	2
MS-related complication	2
Total	8

### Long-term outcome

The mean follow up was 38 months (range 3 to 183 months). Patients were contacted by telephone to assess pain relief or improvement, hypesthesia, and medication use. Five patients were lost to follow up, including four patients with MS.

During follow-up, 28 (65.1%) patients reported complete pain relief without medication (BNI 1). Three patients (7.0%) reported occasional pain attacks without the need for medication (BNI 2). Additionally, ten patients (23.3%) were satisfied with the ongoing use of medication (BNI 3). In summary, 41 of 43 patients (95.3%) reported a BNI score of 1–3 and general long-term satisfaction after surgery. Table 3 and 4 shows the preoperative and last BNI score during FU for facial pain and numbness.

**Table 3:** Comparison of preoperative BNI score for pain and during Follow up and numbness score

	BNI score before PSR (n = 48)	BNI score during Follow up (n = 43)
BNI pain score	pain	pain
1: no pain, no medication	0	28 (65.1%)
2: occasional pain, not requiring medication	0	3 (7.0%)
3: some (3b) or no pain (3a), adequately con-trolled with medication	3 (6.3%)	10 (23.3%) 3a: 4 (9.3%) 3b: 6 (14.0%)
4: some pain. Not adequately controlled with medication	30 (62.5%)	2 (4.7%)
5: severe pain or no relief	15 (31.3%)	0

**Table 4:** BNI numbness score during Follow up

BNI numbness score	Follow up (n = 43)
1: no numbness	10 (23.3%)
2: mild numbness	32 (74.4%)
3: somewhat bothersome facial numbness	1 (2.3%)
4: very bothersome facial numbness	0

In the MS subgroup, nine of the 15 patients reported no pain (8 patients with BNI score 1, 53.3. %) or mild pain without the need of medication (1 patient with BNI score 2, 6.7%) which we rated as excellent results. Two patients did not benefit from PSR during follow-up (BNI score 3, 20%).

We observed 22 patients with recurrent TN after previous MVD who received PSR. The mean time between initial MVD and PSR was 50.1 month. During the mean follow up of 32.4 months after PSR 18 patients (81.8%) still reported good or complete pain relief, 4 of them with the ongoing use of reduced medications during FU. One patient reported no improvement after performing PSR.

One patient received radiosurgery additionally three months after PSR because of persisting pain and improved during follow up without the aid of medication.

The statistical analysis showed a significant improvement in pain compared to the preoperative BNI score during follow-up for all patients (p < 0.0001), including the subgroup with multiple sclerosis (p < 0.0001). There was no significant relation between the degree of pain relief and the extent of rhizotomy in our study. Although some patients reported a mild improvement of their sensory deficit during follow-up, no significant change during follow-up was observed (p < 0.8963).

### Recurrence rate during follow-up

Thirteen patients reported recurrent pain either at the same or adjacent sensory area during FU. In five patients, we performed re-surgery after initial PSR of the lower third. The time interval varied between 5—55 months between first and second PSR. The symptoms returned after 5–48 months. One MS patient described recurrent pain of the same branch. Microsurgical inspection performed 4.6 years after primary PSR surprisingly showed an apparently “intact” nerve. Therefore, PSR of the lower third was repeated with an excellent result. The other four patients described new pain in V2. Therefore, PSR was expanded to approximately 50% and to 2/3 of the sensory nerve root in two patients each with excellent results. All patients with a second look surgery were pain free during follow-up. One patient was offered another PSR of the second branch and newly affected 3rd branch, but refused. Seven out of the thirteen patients with recurrent pain who had not previously responded to medical treatment alone reported satisfaction with medical therapy following PSR. As a result, they declined more invasive treatment options. Only one patient remained dissatisfied and subsequently underwent Gamma Knife surgery.

## Discussion

In our study, patients undergoing PSR experienced long-term pain relief, even in cases of recurrent or treatment-refractory TN. The management of recurrent or treatment-refractory TN remains particularly challenging. Despite previous interventions such as MVD, symptom recurrence remains a significant concern, possibly due to progressive focal demyelination as a potential underlying pathophysiological mechanism [10, 16]. Our findings suggest that PSR can provide effective pain control in carefully selected patients and may serve as a valuable option when other treatments have failed. Although surgical options for refractory TN are limited, patients often seek further intervention. Spatz et al. reported in 2007 that 88% of patients preferred surgical treatment for refractory TN [41].

In our study, there were 15 patients without a microvascular conflict or MS-related TN. Nevertheless, they were looking for advice regarding a surgical treatment option because they were dissatisfied with medical treatment or percutaneous procedures. Symptomatic TN may also be associated with other underlying causes, such as cerebral inflammation, idiopathic intracranial hypertension, or genetic factors [2, 18, 32].

To date, only a few studies with relatively large patient numbers have evaluated the outcomes of PSR [1, 17, 44]. Abhinav et al. compared patient satisfaction after PSR in patients with MS versus non-MS patients [1]. More recent studies included smaller cohorts [8, 13, 15, 28, 29].

In 2017, Jafree et al. assessed the impact of pain and potential complications in 49 patients but did not report five-year recurrence rates. They noted that PSR was associated with higher complication rates and poorer long-term quality of life compared with MVD but both had a negative impact on quality of life [21]. In another survey, Zakrzewska et al. reported less satisfying outcome for patients undergoing PSR, however they also stated that there is no difference in the recurrence rate between MVD and PSR if it was not the first procedure [44].

In 2024, a systematic review and meta-analysis investigated the long-term outcomes of repeat surgical interventions for recurrent or refractory TN, including MVD, stereotactic radiosurgery, and percutaneous rhizotomy, but notably excluded PSR [38]. In our opinion, there has been a lack of large cohorts studying PSR in recent years, particularly in patients without neurovascular conflict. A review from 2007 even suggested that PSR was largely obsolete due to the better tolerability of MVD [42]. While some studies have compared PSR to other treatment options, these mainly involved patients with MS. [1, 4, 21] Only a few case studies or comparative reports exist, e.g. by Díaz-Molina et. al. and Liu et. al. with five patients each [15, 29]. Most modern research has focused on combined approaches (MVD + PSR) rather than PSR as a stand-alone procedure, as highlighted in the systematic review by Montanos and Sousas systematic review and meta-analysis in 2024 [35, 40].

To our knowledge, the present study represents one of the largest cohorts of patients treated with PSR for therapy-refractory or recurrent TN, underscoring the continued relevance of this procedure in selected cases.

### Pain relief

The vast majority of patients in our series reported substantial benefit from PSR, with most achieving complete or near-complete pain relief and others remaining satisfied with continued medication. Overall, 95% of patients experienced meaningful improvement, highlighting the efficacy of PSR in this cohort. In 2007, Tatli compared the pain-free rate of various treatment methods in a literature review. As expected, MVD achieved the best results (65–89%), followed by thermocoagulation (25–82%) and balloon compression (80%). The pain-free rate observed in our PSR cohort is consistent with previously reported outcomes, supporting the efficacy of PSR in selected patients [42].

Other studies compared the success rate of MVD vs MVD + PSR. Zhang et al. found a slightly higher pain-free percentage during follow up in patients with MVD + PSR [45]. Those results were confirmed by Chen et al. with their systematic review and meta-analysis of 8172 patients[9]. Although, a further meta-analysis in 2024 found no advantage of a combined MVD + PSR approach in long-term pain improvement or recurrence rate compared to MVD alone if there is no neurovascular conflict [35]. In their systematic review and meta-analysis from 2024, Sousa et al. also reported no differences in long-term outcomes and recurrence rates in 1,011 patients treated with MVD and 327 with MVD + PSR. The combined group reported greater satisfaction with pain relief only during the immediate postoperative period; however, a higher incidence of hypesthesia was observed [40].

In our opinion, MVD + PSR in neurovascular compression is not conclusive. Either there is a neurovascular conflict that can be treated by MVD, or there is not. Additional PSR means sacrificing a nerve which might not be necessary.

Our suggestion in pain recurrence after MVD includes re-exploration, removal of the Teflon sponge or re-decompression in case of persistent vascular compression, or finally PSR.

### Complications and hypesthesia

A frequently cited concern regarding PSR is its purportedly higher rate of complications. Reports range from hypesthesia and hearing loss to serious complications such as anesthesia dolorosa, meningitis and Herpes simplex virus (HSV) infection. Gao et al. even reported a complication rate of 52.3% following PSR [17]. Needless to say, postoperative hypesthesia is an expected result due to the surgical sectioning of sensory fibers. In our cohort, most patients are not bothered by the sensory loss. If patients have been adequately informed about hypesthesia before the operation, we do not consider this to be a complication but an expected result. Only two patients in this study described the sensory deficit as mildly disturbing right after surgery and negated any problems during FU. The sensory loss was very variable but always attributed to the sacrificed branch. Interesting and still not understood is a fully intact sensation after PSR which we could observe occasionally.

The complication rate of hearing impairment in the literature for PSR is up to 25% [42]. The risk of reactivation of HSV after PSR and postoperative Herpes labialis is ranging from 1 to 10%, especially in patients with known recurrent herpes labialis or recurrent surgery. Klun who even described a Herpes labialis rate of nearly 100% after PSR, suspected a correlation between the extent of nerve manipulation and the occurrence of herpes labialis [7, 25, 26]. We have no comparative values, as these complications did not occur in our series. We just observed transient hearing impairment with full recovery in two patients.

In the literature, the higher complication rate reported after PSR has often been cited as an argument against its use. However, our experience challenges this perspective. Performing PSR under direct visualization allows for precise tailoring of fiber sectioning based on each patient’s specific pain distribution. This approach offers greater control compared to percutaneous thermocoagulation. Percutaneous procedures or radiosurgery are also associated with post-interventional hypesthesia, but this fact is rarely used as an argument against these procedures.

Although previous series have reported complications such as corneal anesthesia and anesthesia dolorosa following PSR, no such events were observed in our cohort. Nevertheless, these potential risks should be kept in mind when counselling patients and planning surgery [1, 20, 26, 37].

### Recurrence rate

A pain recurrence rate of 10% to 49% after five years, affecting the same or different branches, has been reported following PSR [4, 9, 28, 42–44, 46]. Our observed recurrence rate of 27.1% is consistent with these findings. A meta-analysis by Chen et al. reported a recurrence rate of 20.9% for gamma-knife surgery, 12.3% for percutaneous balloon compression, and 11.9% for thermocoagulation [9]. The recurrence rate for percutaneous procedures in particular varies greatly. Tatli et al. reported a range of 18.1% to 80% for thermocoagulation. Following glycerol injection recurrent pain also occurred in 41 to 81% of cases [42]. Therefore, the recurrence rate of PSR seems to be acceptable.

## Limitations

The study is still limited due to the small number of patients. Furthermore, it is a single center study. The extent of individual PSR was just estimated by two senior surgeons. Finally, the non-comparative design precludes direct comparisons with other treatment modalities.

## Conclusion

Our results confirm that PSR is a viable option for therapy-refractory or recurrent TN. Even if the patient had undergone multiple other treatments previously without satisfactory pain relief, PSR has proven to be effective. Patients with multiple sclerosis, in particular, may represent a group that should be informed early about PSR as a treatment option, because 66% of these patients reported total or partial pain relief. Even after successful MVD and recurrent pain, PSR should be discussed as an option if there is no persistent vascular compression.

In our opinion, PSR should be considered particularly in MS patients and patients who do not prefer recurrent percutaneous procedures or who have not experienced improvement with those interventions. Adequate patient counseling regarding the expected hypesthesia is important.

The treatment of recurrent or refractory TN remains challenging, but PSR should be considered as a surgical alternative in those patients.

## Data Availability

No datasets were generated or analysed during the current study.
